# Augmentation of soft tissue volume at pontic sites: a comparison between a cross-linked and a non-cross-linked collagen matrix

**DOI:** 10.1007/s00784-020-03461-8

**Published:** 2020-07-27

**Authors:** Nadja Naenni, Prisca Walter, Christoph H. F. Hämmerle, Ronald E. Jung, Daniel S. Thoma

**Affiliations:** 1 Clinic of Reconstructive Dentistry, Center of Dental Medicine, University of Zurich, Zurich, Switzerland; 2 Department of Periodontology, Research Institute for Periodontal Regeneration, College of Dentistry,, Yonsei University, Seoul, South Korea

**Keywords:** Collagen matrix, Soft tissue, Soft tissue augmentation, Grafting, Dental implants

## Abstract

**Aim:**

To assess histopathological and histomorphometric outcomes of soft tissue volume augmentation procedures at pontic sites using a volume-stable cross-linked collagen matrix (VCMX) and a non-cross-linked collagen matrix (XCM).

**Materials and methods:**

In twelve adult beagle dogs, the mandibular premolars and first molar were hemisected and the mesial root extracted. Soft tissue augmentation was randomly performed using VCMX, XCM, or a sham-operated control. Sacrifice was performed after 4, 8, and 26 weeks. Non-decalcified sections were analyzed for histopathologic and histomorphometric measurements at four different levels below the crest (1.5, 2.5, 3.5, and 5.5 mm).

**Results:**

Group VCMX presented a greater overall amount of soft tissue at all healing time points, more pronounced fibroblast ingrowth, vascularization, and a substantial new collagen deposition. Over time, group XCM demonstrated faster signs of degradation compared with group VCMX. Four weeks after augmentation, group VCMX yielded a higher mean ridge width compared with groups XCM (2.22 mm VCMX, 0.89 mm XCM (at 2.5 mm); 2.05 mm VCMX, 0.80 mm XCM (at 3.5 mm) *p* < 0.05) and sham (0.59 mm sham (at 1.5 mm); 0.48 mm (at 2.5 mm); 0.44 mm (at 3.5 mm) *p* < 0.05). After healing periods of 8 and 26 weeks, measurements in group VCMX remained significantly higher compared with group sham both at 8 weeks (levels of 1.5 mm, 2.5 mm and 5.5 mm) and at 26 weeks (levels of 1.5 mm, 3.5 mm and 5.5 mm) (*p* < 0.05).

**Conclusion:**

The use of a cross-linked collagen matrix resulted in a greater and more stable ridge width over time compared with control groups.

**Clinical relevance:**

Soft tissue volume augmentation at pontic sites is more effective when using a cross-linked compared with a non-cross-linked collagen matrix.

## Introduction

Resorption and remodeling processes of the ridge after tooth extraction cannot be circumvented and often result in contour and volume deficits after healing [1]. In order to re-establish the ridge contour, to optimize esthetics, and to simplify cleanability, soft tissue grafting procedures were proposed at future implant and pontic sites [2–4]. Soft tissue augmentation procedures are therefore performed to improve the tissue quality (gain of keratinized tissue) or the tissue quantity (volume increase). Traditionally, autogenous grafts harvested from the patient’s palate represent the gold standard [5]. The main disadvantage of these procedures is represented by the second surgical site which is the main cause for post-surgical bleeding, increased treatment time, and increased patient morbidity [6–8]. Consequently, the development of substitute materials serving as alternatives for autogenous grafts has been the focus of research over the past 10 years. Recent clinical studies demonstrated that collagen-based matrices can serve as an alternative to autogenous tissue grafts for augmentation of both the quality and the quantity of the soft tissues [9, 10]. These materials are considered a valuable treatment alternative [9, 11]. Soft tissue volume augmentation at pontic and implant sites appears to be a challenging clinical situation with a clear need for a soft tissue substitute. So far, no scientific evidence exists comparing the two most common collagen matrices (with and without cross-linking) for contour augmentation at pontic sites.

The aim of the present study was to assess histopathologic and histomorphometric outcomes of soft tissue augmentation procedures at pontic sites randomly using a volume-stable cross-linked collagen matrix (VCMX), a non-cross-linked collagen matrix (XCM), or a sham-operated control.

## Materials and methods

### Study design

The study was designed as a randomized controlled experimental study employing 12 female beagle dogs at the age of 16–20 months, weighing 7–12.8 kg. The dogs were housed under monitored laboratory conditions conforming with the European requirements (EEC/2010/63). Dogs were fed a soft diet during the entire study period. The study protocol was approved by the local ethical committee of NAMSA (Lyon, France) 189,735 and conducted in accordance with the OECD Good Laboratory Practice regulations, ENV/MC/CHEM (98) 17, with the European Good Laboratory Practice regulations, 2004/10/EC Directive and with the United States Food and Drug Administration Good Laboratory Practice regulations, 21 CFR 58.

### Surgical interventions

All surgical procedures were performed under general anesthesia and under sterile conditions by two surgeons (DT, NN) in an operating room. Premedication included antibiotics (Buccoval, Sogeval, and Duplocilline, Intervent S.A) and pain relief medication (Dorbene Vet, Zoetis, and Buprecare, Axience). Prior to the surgery, the respective hemimandible was disinfected with 0.2% chlorhexidine solution and the region was locally anesthetized using lidocaine hydrochloride with adrenaline (Ubistesin forte, 3 M ESPE or Lidocaine adrenaline, Aguettant). The respective surgical procedures and the applied medication were described in detail in a previous publication (Thoma et al., 2017).

### Extractions

Two premolars (P3, P4) and the first molar (M1) were treated in each hemimandible of the dogs. The mesial roots were carefully extracted, and the distal roots’ root-canal treated.

### Soft tissue augmentation

**Fig. 1:**
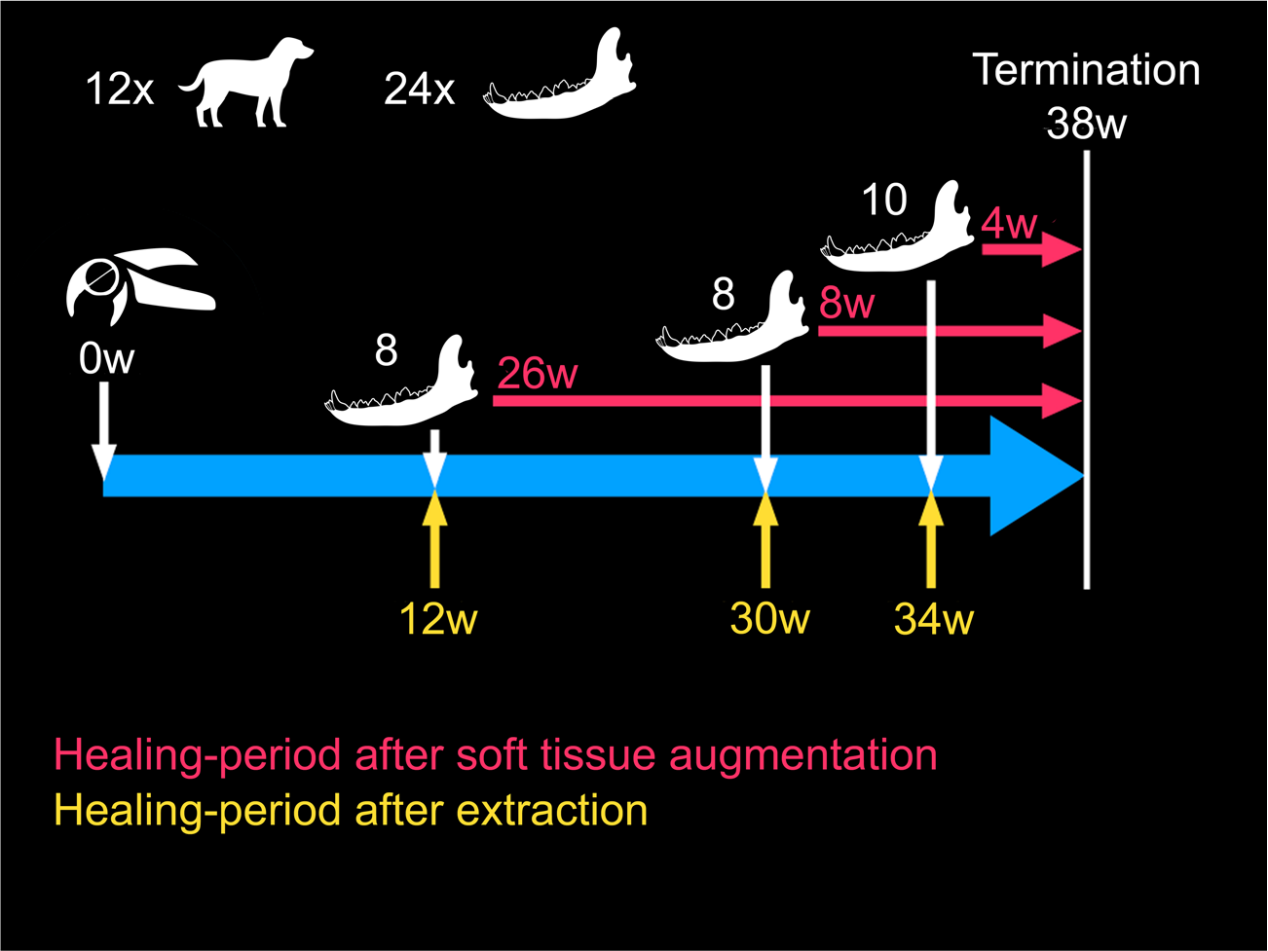
Timeline of the entire study period with the respective healing periods for elapsed time after tooth extraction and time after soft tissue augmentation procedures

After tooth extraction, soft tissue augmentation surgery was performed at predefined healing periods (12, 30, or 34 weeks) (Fig. 1). A mid-crestal full-thickness incision extending from M1 to P4 and P3, respectively, as well as sulcular incisions at the remaining roots (M1, P3, P4) were performed. Split-thickness flaps were prepared, creating a pouch at the buccal aspect of each site. Subsequently, the following three treatment modalities were applied according to a predefined computer-generated randomization table:

- VCMX, cross-linked volume-stable cross-linked porcine collagen matrix (Fibro-Gide®, Geistlich Pharma AG, Wolhusen, Switzerland)
- XCM, non-cross-linked porcine collagen matrix (Mucograft®, Geistlich Pharma AG, Wolhusen, Switzerland)
- Sham, incision and flap elevation without soft tissue augmentation

**Fig. 2:**
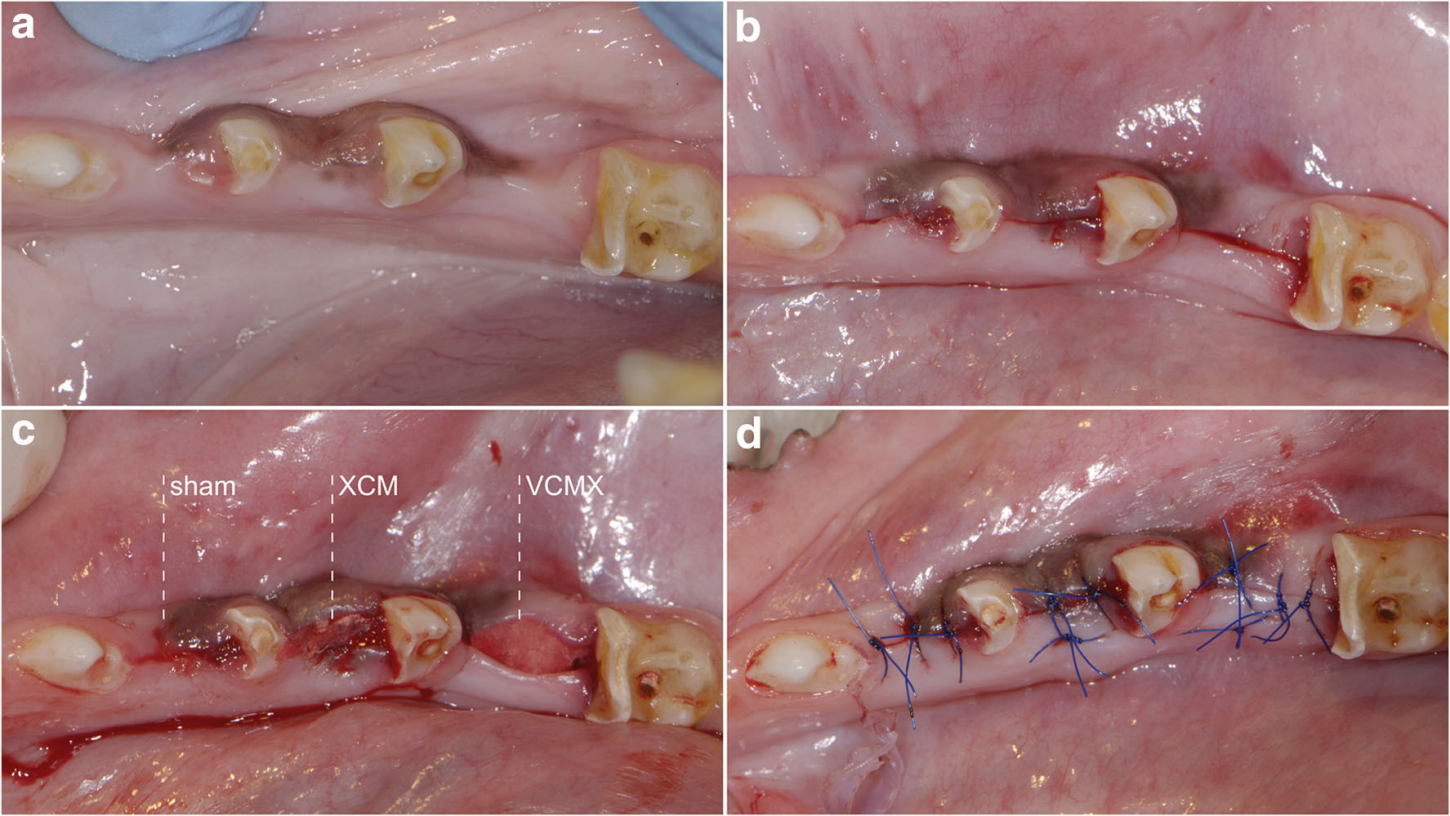
Soft tissue augmentation procedure. **a** Clinical situation after initial healing period after tooth extraction; **b** preparation of the buccal pouch; **c** soft tissue augmentation procedure for the respective groups: sham, control (XCM), and test (VCMX). **d** clinical situation after primary wound closure. Note the lingual suture in groups control and test to fixate the graft to the lingual

The dimension of both the VCMX and XCM were adapted in length and width according to the size of the defect. A thickness of 5 mm was ensured for both substitute products prior to insertion. Both matrices were placed buccally in the prepared pouch. Immobilization was ensured by applying a single horizontal mattress suture at the lingual aspect. Subsequently, primary and tension-free wound closure was obtained by applying one horizontal mattress suture at the center of the site. Additional single-interrupted sutures were placed mesially and distally thereof (Fig. 2).

In order to end up with the predefined healing periods, soft tissue augmentation in each dog was performed at two different time-points (one per hemimandible) following the above described protocol (Fig. 1).

After the surgical interventions, the dogs were fed a soft diet and received antibiotics once a day (Buccoval, Sogeval) as well as anti-inflammatory drugs (Carprodyl, CEVA) and local disinfection (CHX, Cooper) until suture removal 14 days later. Thirty-eight weeks after tooth extraction/devitalization, all dogs were euthanized by a lethal injection of a barbiturate (Dolethal, Vetoquinol S.A., Paris, France). This corresponded to 4, 8, and 26 weeks of healing (after the tissue augmentation) (Fig. 1).

### Histological preparation

After initial fixation and dissection of the hemimandibles (cut with a band saw into one block per site), each block was additionally fixated in 10% neutral buffered formalin. The blocks were dehydrated in alcohol solutions of increasing concentration, cleared in xylene, and embedded in polymethylacrylate. After transversal laser-sectioning into 4 samples (LLS ROWIAK LaserLabSolutions GmbH, Hannover, Germany), each block was stained with McNeal and afterward EXAKT cutting was performed (approximately 30–40 um thick) and the obtained slides were thereafter analyzed.

### Histopathologic and histomorphometric analysis

**Fig. 3:**
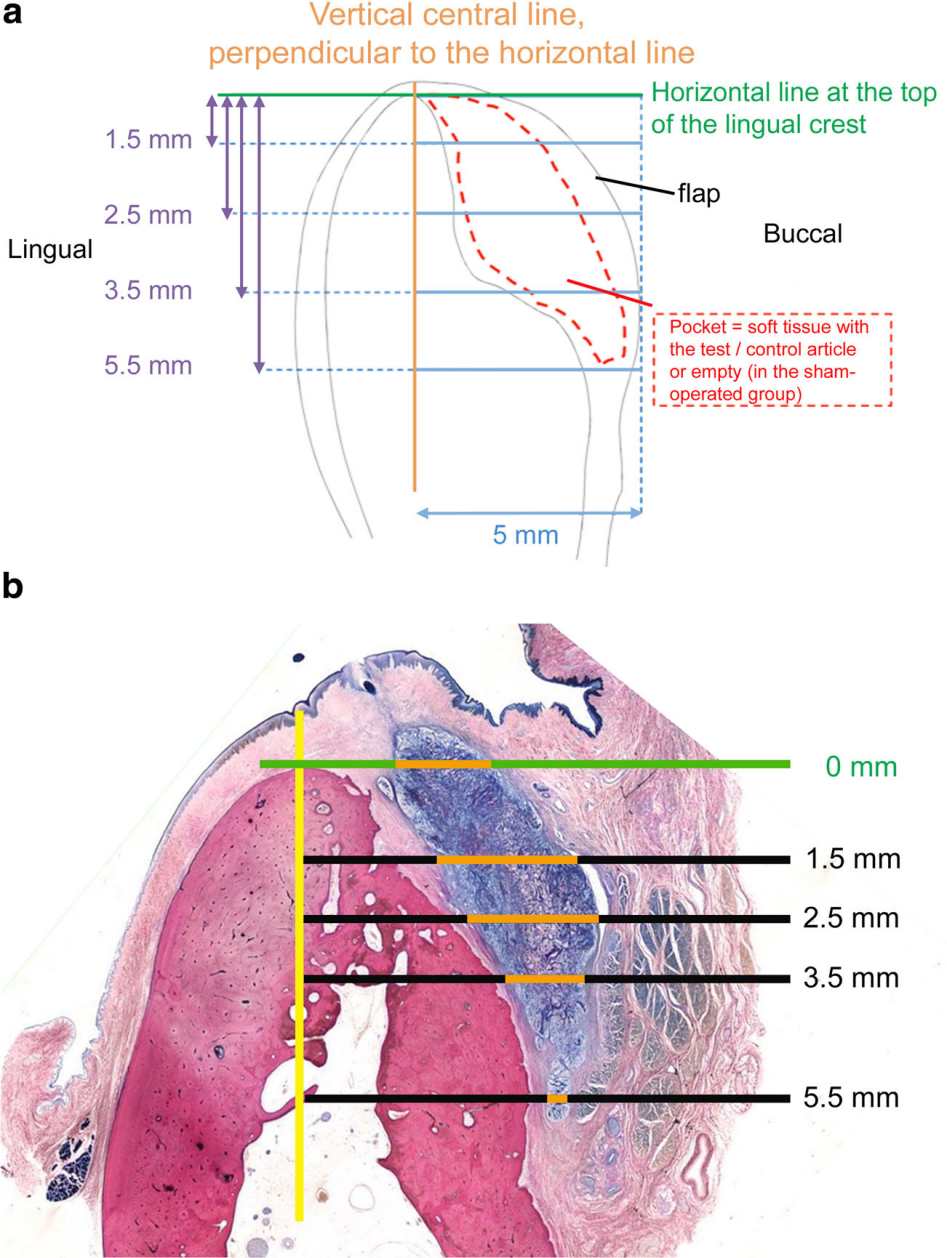
**a** Histomorphometric analysis. A vertical line (yellow) and a horizontal line (green) were drawn. Four additional horizontal lines (blue) were drawn at different levels (1.5, 2.5, 3.5, and 5.5 mm) covering the entire augmented area. The ridge width was measured along these five lines for mineralized tissue, native soft tissue, and augmented soft tissue. **b** Superimposition of the schematic illustration on a histologic slide to provide an overview of the measurements carried out. The orange part of the line represents the augmented soft tissue

A qualitative and semi-quantitative evaluation of the histologic sections under light microscopy (Nikon Eclipse 80i, Nikon, Tokyo, Japan) was performed in adaptation to ISO 10993-6 and in compliance with the standard nomenclature of the International Society for Stereology. For the histomorphometric outcomes assessment, the borders of the prepared pouch were defined for each group. A vertical line (corono-apical axis) and a horizontal line (bucco-lingual axis) perpendicular to it were drawn from the top of the lingual crest. Four additional horizontal lines were drawn at different levels (1.5, 2.5, 3.5, and 5.5 mm) covering the entire augmented area. The ridge width was measured along these five lines for both bone tissue and augmented soft tissue (Fig. 3).

### Statistical analysis

**Fig. 4:**
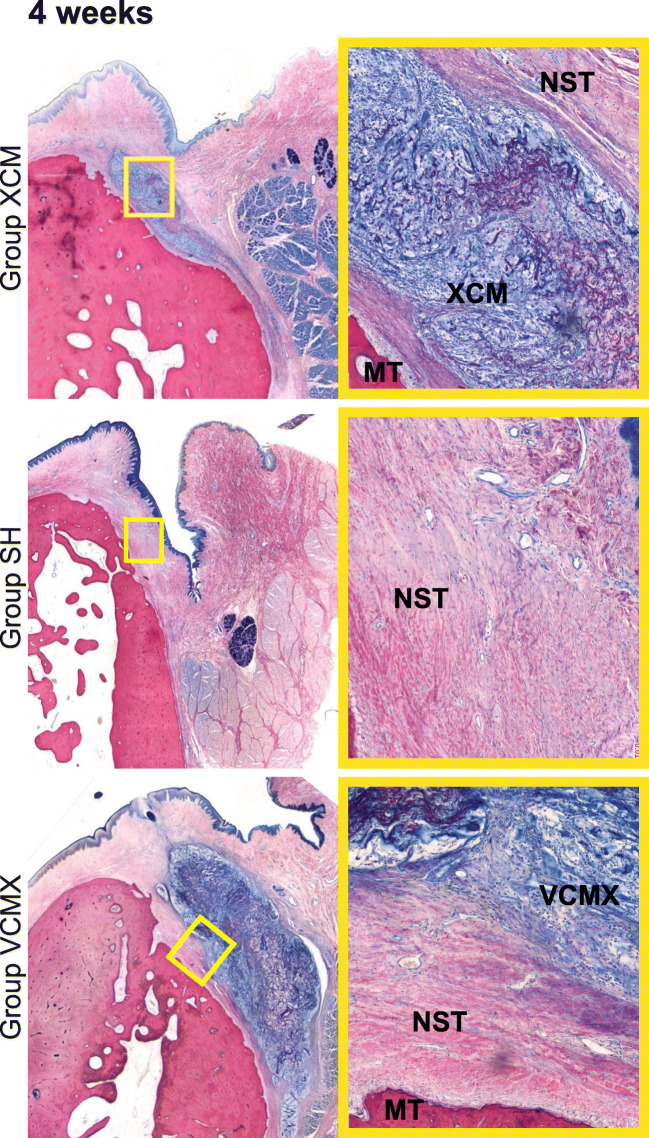

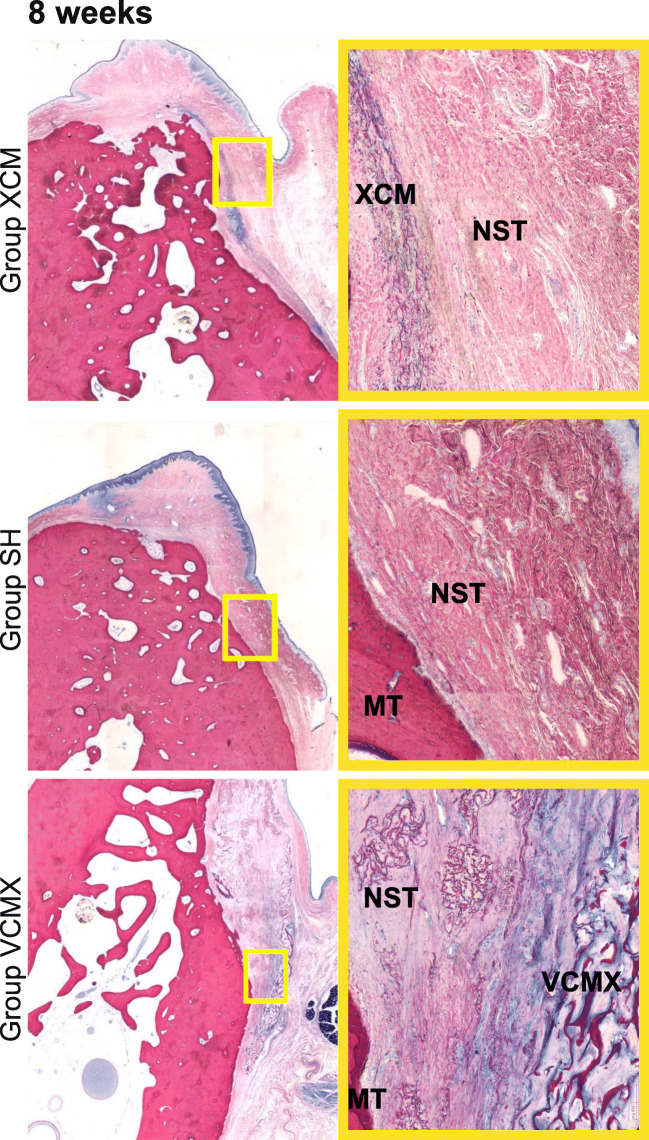

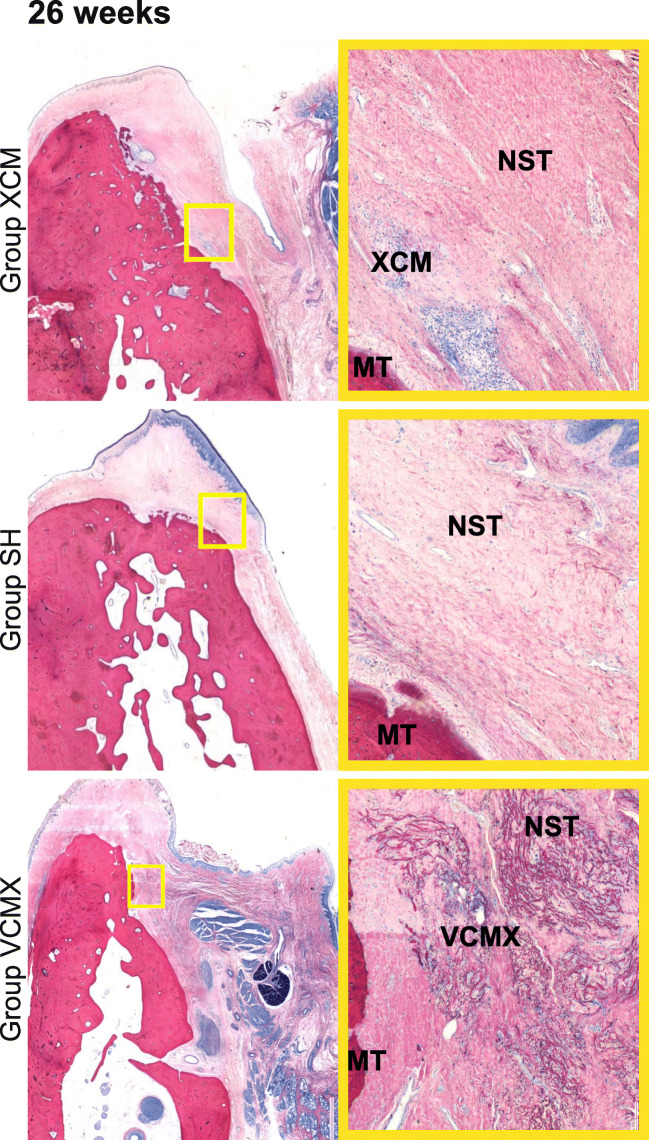
Histologic slides showing the three groups (XCM, sham, and VCMX) at different healing periods of 4, 8, and 26 weeks. All slides stained with McNeal. High-resolution images on the right representing the center of the augmented area (× 10 magnification). MT, mineralized tissue; NST, native soft tissue; XCM, non-cross-linked collagen matrix; VCMX, volume-stable cross-linked collagen matrix

Data were summarized in terms of mean, standard deviation, and confidence interval. Macroscopic as well as the qualitative and semi-quantitative histopathologic inflammatory parameters were compared between the investigated groups. For each group, comparisons between the different healing periods (4, 8, and 26 weeks) (Fig. 4) were calculated. Histopathologic inflammatory parameters were described descriptively. Based on the histopathologic and histomorphometric evaluation, a statistical analysis was conducted (ANOVA (5% risk) applying a statistical software (Software SSPS Version 24.0, IBM, Zurich, Switzerland).

## Results

### Clinical and macroscopic findings

During the entire study period, the dogs remained healthy and no systemic complications or local intolerances at the augmented sites occurred.

### Descriptive histology

After a healing period of 4 weeks, group VCMX demonstrated a higher amount of soft tissue at the augmented site compared with group XCM. In addition, more pronounced fibroblast ingrowth and vascularization as well as a substantial new collagen deposition were observed in group VCMX compared with group XCM. At 8 and 26 weeks of healing, group VCMX presented with a greater overall amount of soft tissue compared with XCM, while in both groups, a material degradation had occurred. Over time, group XCM showed faster signs of degradation compared with group VCMX. The sham group healed without local inflammation and demonstrated no visible soft tissue augmentation at all time-points.

### Histomorphometric analyses

Four weeks after soft tissue augmentation, group VCMX yielded a higher mean amount of augmented soft tissue compared with groups XCM and sham at all measured levels. The differences between VCMX and XCM (2.22 mm VCMX, 0.89 mm XCM (at 2.5 mm); 2.05 mm VCMX, 0.80 XCM (at 3.5 mm)) and between VCMX and sham (2.24 mm VCMX, 0.59 mm sham (at 1.5 mm); 2.22 mm VCMX, 0.48 mm sham (at 2.5 mm); 2.05 mm VCM, 0.44 mm sham (at 3.5 mm)) reached statistically significant differences (*p* < 0.05). At 8 weeks, group VMCX still yielded a greater amount of augmented soft tissue compared with group XCM at all levels without reaching statistical significance. Compared with the sham group, values measured at 1.5 mm (1.17 mm VCMX, 0.43 mm sham), 2.5 mm (1.15 mm VCMX, 0.48 mm sham), and 5.5 mm (0.81 mm VCMX, 0.29 mm sham) reached statistical significance in favor of group VCMX (*p* < 0.05). At 26 weeks of healing, group VCMX had greater values of augmented soft tissue at all measured levels compared with group XCM without reaching statistical significance. Compared with group sham, group VCMX reached significantly higher values (*p* < 0.05) (1.11 mm VCMX, 0.39 mm sham (at 1.5 mm); 1.04 mm VCMX, 0.47 mm sham (at 3.5 mm);0.90 mm VCMX, 0.14 mm sham (at 5.5 mm)) except for the level of 2.5 mm (Table 1).

**Table 1:** Histomorphometric analysis—horizontal measurements

Time period	Group		0 mm			1.5 mm			2.5 mm			3.5 mm			5.5 mm		
			mineralized tissue	native soft tissue	augmented soft tissue	mineralized tissue	native soft tissue	augmented soft tissue	mineralized tissue	native soft tissue	augmented soft tissue	mineralized tissue	native soft tissue	augmented soft tissue	mineralized tissue	native soft tissue	augmented soft tissue
4 W	**XCM** (*n* = 8)	**Mean** SD	**0.0** 0.0	**1.19** 0.42	**1.64** 0.82	**1.64** 0.83	**1.12** 0.91	**1.11** 0.70	**2.28** 0.64	**1.15** 0.66	0.89* 0.47	**2.81** 0.40	**0.99** 0.28	0.80* 0.31	**3.20** 0.30	**1.01** 0.37	0.78* 0.24
	**SH** (*n* = 8)	**Mean** SD	**0.0** 0.0	**1.74** 0.89	**1.20** 0.55	**1.95** 0.54	**1.24** 0.72	0.59* 0.19	**2.47** 0.57	**1.35** 0.66	0.48* 0.20	**2.76** 0.78	**1.38** 0.54	0.44* 0.36	**3.15** 0.60	1.39* 0.50	0.44* 0.24
	**VCMX** (*n* = 8)	**Mean** SD	**0.0** 0.0	**1.14** 0.68	**2.34** 1.17	**1.20** 0.58	**0.98** 0.86	2.24* 1.20	**1.76** 0.65	**0.92** 0.76	2.22* 1.18	**2.21** 0.70	**0.74** 0.68	2.05* 0.99	**3.20** 0.34	0.61* 0.51	**1.20** 0.68
8 W	**XCM** (*n* = 8)	**Mean** SD	**0.0** 0.0	**1.44** 0.68	**1.09** 0.43	**1.51** 0.59	**1.01** 0.49	**0.90** 0.54	**2.02** 0.54	**1.82** 0.76	**0.78** 0.28	**2.46** 0.48	**1.66** 0.69	**0.68** 0.20	**3.32** 0.46	**1.11** 0.67	**0.41** 0.23
	**SH** (*n* = 8)	**Mean** SD	**0.0** 0.0	**2.04** 0.63	**0.53** 0.64	**1.65** 0.49	**1.46** 0.98	0.43* 0.13	**1.91** 0.52	**1.63** 1.16	0.48* 0.17	**2.26** 0.66	**1.66** 0.90	**0.51** 0.28	**3.07** 0.35	**1.54** 0.83	0.29* 0.21
	**VCMX** (*n* = 8)	**Mean** SD	**0.0** 0.0	**1.57** 0.87	**1.00** 0.74	**1.30** 0.60	**1.00** 0.74	1.17* 0.37	**1.94** 0.63	**1.16** 0.78	1.15* 0.51	**2.43** 0.78	**1.04** 0.73	**1.05** 0.61	**3.03** 0.51	**1.16** 0.60	0.81* 0.47
26 W	**XCM** (*n* = 8)	**Mean** SD	**0.0** 0.0	**1.59** 0.88	1.30* 0.90	**1.67** 0.52	**1.31** 0.89	**0.83** 0.28	**2.21** 0.54	**1.10** 0.81	**0.64** 0.22	**2.45** 0.51	**1.25** 0.76	**0.69** 0.45	**3.22** 0.47	**1.02** 0.69	**0.57** 0.42
	**SH** (*n* = 8)	**Mean** SD	**0.0** 0.0	2.54* 0.71	0.20* 0.49	**1.63** 0.68	**1.70** 0.97	0.39* 0.19	**2.00** 0.50	**1.45** 0.96	**0.62** 0.26	**2.56** 0.43	**1.50** 0.60	0.47* 0.17	**3.06** 0.18	1.62* 0.50	0.14* 0.14
	**VCMX** (*n* = 8)	**Mean** SD	**0.0** 0.0	0.74* 0.25	1.86* 0.21	**1.43** 0.31	**0.90** 0.59	1.11* 0.60	**1.93** 0.31	**1.35** 0.70	**1.20** 0.57	**2.51** 0.45	**1.24** 0.52	1.04* 0.35	**3.25** 0.37	0.79* 0.35	0.90* 0.46

All mean-values are bold

Statistically significant values are marked with a *

## Discussion

The present preclinical study analyzing histologic and histomorphometric outcomes following soft tissue augmentation applying two collagen matrices predominantly revealed (i) a faster integration, vascularization, and fibroblast ingrowth of VCMX at the earliest time-point (4 weeks) compared with XCM; (ii) a more pronounced degradation at 8 and 26 weeks in group XCM compared with group VCMX; and (iii) a higher mean ridge width for group VCMX at all time-points compared with groups XCM and sham.

Soft tissue volume augmentation is a frequently performed muco-gingival procedure applied at pontic as well as at implant sites. Due to an increased rate of patient morbidity when using the gold standard, the autogenous soft tissue graft, research has focused on soft tissue substitutes. Such devices need to fulfill a number of criteria: fast integration into the surrounding tissues with a minimal inflammatory reaction, degradation and replacement by autogenous soft connective tissue, and sustained stability of the augmented site. The observed inflammatory reaction in the present study was minimal in all groups. The histologic analysis showed stable remodeling processes and integration of both substitute materials over time. Similar healing and comparable histological outcomes were also shown in a previous clinical study comparing XCM with a free gingival graft in an open-healing approach [12]. Another previously published pre-clinical study demonstrated a complete replacement of XCM by healthy connective tissue within 30 days, leading to an optimal integration of the substitute material [13]. In the present study, vascularization and fibroblast ingrowth were more pronounced in sites augmented with the cross-linked matrix at 4 weeks. Furthermore, a substantial new collagen deposition was observed in this group which subsequently led to a more pronounced gain of the augmented soft tissue area. In the non-cross-linked collagen matrix groups, tissue integration was delayed compared with group VCMX. The cross-linked structure of the investigated matrix seems to better mimic a scaffold promoting increased cell-ingrowth compared with the non-cross-linked XCM if applied in a submerged setting. Integration of the cross-linked collagen matrix after 1 month was also shown in previous preclinical studies [3, 14]. It was shown that the degree of cross-linking of the collagen-based matrix correlated with an improved tissue integration. Not only did previous investigations demonstrate favorable tissue integration of the cross-linked matrix but also increased vascularization and connective tissue formation [3, 15]. Comparing an autologous connective tissue graft to the VCMX in a clinical study, histological analysis revealed good integration of the cross-linked collagen matrix [11].

Along with a deposition and formation of collagen fibers within the network structure of the two matrices, both soft tissue substitutes demonstrated variable degrees of degradation depending on the time-point and matrix. At 4 weeks, degradation was more pronounced in both collagen matrix groups, whereas thereafter more stable remodeling processes were observed. In addition, the group VCMX demonstrated a less distinct degradation and a higher stability of the network structure compared with groups XCM. This is in line with results from prior preclinical studies demonstrating fast degradation of non-cross-linked collagen membranes [16, 17]. The difference in network structure between the two investigated collagen matrices has an influence on degradation time, mechanical stability, and connective tissue ingrowth [5, 14] . Both substitute materials have been specifically developed for different clinical indications. XCM is predominantly indicated for gain of keratinized tissue and can be left exposed during healing, even though the matrix has been applied for gain of soft tissue thickness in various other indications. Since the main indication, however, is an open-healing approach, the matrix is non-cross-linked. In contrast, VCMX is built up by cross-linked collagen and is indicated for procedures aiming at volume gain. Due to its porous structure, VCMX is supposed to be placed in a submerged approach. Furthermore, the cross-linked matrix facilitated early vascularization and demonstrated network presence after 24 weeks [18]. This is in line with the present study. Over time, remodeling and degradation of the matrices was observed. These processes led to a decrease of the augmented area between 2 and 6 months after augmentation both for VCMX and autologous connective tissue grafts in a preclinical setting [3]. This is supported by contour measurements, resulting in a volume decrease for VCMX as well as for autogenous connective tissue grafts in a preclinical study [19]. Results from the present study revealed a decrease of the obtained soft tissue amount mainly within the first 8 weeks (roughly 50%). This leads to the speculation that an over-augmentation of the soft-tissue may be beneficial regarding the anticipated decline of the substitute material. There is, however, no scientific evidence whether or not such an over-augmentation would result in more favorable outcomes.

The histomorphometrically assessed ridge width at the three time-points did not result in an increased ridge width at all time-points in the sham group. Groups XCM and VCMX revealed higher values at 4 weeks compared with 8 and 26 weeks. Moreover, ridge width values were in favor of group VCMX at all time-points. A randomized clinical trial revealed that an autogenous connective tissue graft was more effective than a non-cross-linked collagen matrix for improving soft tissue thickness [20]. When comparing the clinical use of a cross-linked collagen matrix with an autologous connective tissue graft, the substitute material performed similar regarding soft tissue volume increase at implant sites [21]. These results might be explained by the difference in structure of the cross-linked matrix. This provides an improved leading structure for fibroblast ingrowth compared with a non-crossed-linked matrix. The outcomes of the present study are in line with the results from an earlier clinical study in which the VCMX performed well and even surpassed the gold standard connective tissue graft regarding buccal volume augmentation [11]. From a clinician’s and patient’s perspective, harvesting an autologous connective tissue graft from the palate is time-consuming and associated with an increased patient morbidity. The use of a substitute material is associated with a shorter surgical time and decreased patient morbidity [20]. In terms of patient-related outcomes, collagen matrix as a substitute material seems to be a viable alternative in order to avoid this negative effect.

## Conclusions

The use of a volume stable cross-linked collagen matrix resulted in a greater and more stable ridge width over time compared with a non-cross-linked collagen matrix when applied in a submerged approach. Tissue integration as assessed by fibroblast ingrowth was more favorable for the cross-linked collagen matrix compared with the non-cross-linked collagen matrix.
